# The Role of Extended Family Members in the Lives of Autistic Individuals and Their Parents: A Systematic Review and Meta-Synthesis

**DOI:** 10.1007/s10567-025-00525-7

**Published:** 2025-05-20

**Authors:** Jia-Ling Li, Melissa Washington-Nortey, Tsegereda Haile Kifle, Francesca Cotier, Rosa A. Hoekstra

**Affiliations:** Department of Psychology, Institute of Psychiatry, Psychology and Neuroscience, King’s College London, London, UK

**Keywords:** Autism, Extended family members, Grandparents, Parents, Support, Stigma

## Abstract

Extended family members play an important role in meeting the care needs of autistic individuals, yet family support policies and practices often overlook this role. We aimed to synthesise qualitative research on the role played by extended family members in the lives of autistic individuals and their parents and identify cultural patterns. We searched eight databases and selected relevant studies through a two-stage screening process. We synthesised the results and discussions described in the selected studies using template analysis. The review included 42 studies (40 qualitative; 2 mixed methods), reporting on 1048 parents and 2140 grandparents. While aunts, uncles, and cousins were not direct participants, their roles were described in participants’ narratives. Three main themes were developed: (1) types of support from extended family members, including emotional, financial, instrumental, and informational support; (2) unhelpful or lack of support from extended family members, including misunderstanding about autism, absence of support with caregiving, and negative attitudes and discriminatory behaviours against autistic individuals as well as their parents; (3) factors influencing the role of extended family members, including individuals, family unit, family interaction characteristics, and a journey towards acceptance and cultural influences. Novel findings on the influence of culture suggested in cultures highly valuing family interdependence, extended relatives play a more prominent support role, yet the emphasis on family reputation might hinder the acceptance of autism by extended relatives. Based on a family systems approach, we recommend priorities for intervention development and clinical practice to support the effective involvement of extended family members.

**Supplementary Information:**

The online version contains supplementary material available at 10.1007/s10567-025-00525-7.

## Introduction

Autism is a neurodevelopmental disability characterized by difficulties in social communication and behaviours throughout the lifespan, affecting about 78 million people and their families worldwide (Lord et al., 2022). Extended family members, including grandparents, uncles, aunts, and cousins, are often a key part of the support network of families (Alburez-Gutierrez et al., 2023; Treleaven, 2023). However, predominant approaches to promoting family support for autistic individuals and their parents are based on a nuclear family model, often overlooking the involvement of caregivers within the broader family system (Cridland et al., 2014). Consequently, there is a critical gap in understanding the diverse and often culturally shaped roles of extended family members in supporting autistic individuals and their parents across global contexts.

Research to date has largely suggested service provision agencies adopt a family-centred approach to support autism families (Galpin et al., 2018); one that recognises families as a constant in the child’s life and emphasises aligning services with the needs of the whole family (Gabovitch & Curtin, 2009). The family-centred approach taken by services is strongly informed by family system theory, as it recognises the interrelatedness of family members and the importance of considering the needs of the entire family, rather than focussing solely on the child (Turnbull et al., 2010). This framework outlines key aspects of family dynamics, including characteristics, interactions, functions, and life cycle stages. Among its four core subsystems, the extended family subsystem plays an important role contributing to the overall family functioning (Turnbull et al., 2010). Parents of autistic children often face challenges, such as managing their child’s difficulties, financial strain, and social stigma (Ooi et al., 2016). Compared to parents of typically developing children or those with other neurodevelopmental disabilities, they report lower level of health and quality of life (Barroso et al., 2018; Vasilopoulou & Nisbet, 2016). Support from extended family members may help parents better cope with these challenges. However, the current definition of family often narrowly concentrates on families from Western Educated Industrialized Rich and Developed (WEIRD) nations, privileging the nuclear family model, typically composed of biological parents and children (Perez-Brena et al., 2022). As a result, previous reviews on family support for families with autistic children were mostly focussed on parent–child dyads, parent couples, and siblings (Karst & Van Hecke, 2012).

Extended relatives’ involvement in childcare can differ across cultures. Similar to Aubel and Chibanda (2022)’s work on the role of grandparents, Weidman’s framework of health culture guided our understanding of culture. According to Weidman (1998, p.272), health culture includes two dimensions: (1) cognitive and conceptual aspects, such as knowledge, attitudes, and practices related to health; and (2) the social system in which health issues are embedded, including the roles and influence of family, community, and social networks on health status and behaviours. This systematic review considers both dimensions, specifically, cultural influences on knowledge, attitudes, and practice related to autism, and the cultural norms of parenting within the family system. Limited awareness and misconception may shape how extended relatives respond to autism diagnosis. These knowledge-related factors intersect with culturally defined family roles and caregiving expectations. In societies where family life is structured around interdependence and collective responsibility, extended family members are highly important in caregiving and decision-making (Hofstede, 2011; Rogoff, 2003). For instance, in many areas of Africa, Asia, and Latin America, it is common to live in extended family household (Kamiya & Hertog, 2020), or share childcare responsibilities with extended relatives. The limited attention given to extended family members may restrict understanding of family-centred support needs and provisions and prevent service providers from acknowledging the importance of their role in the care of autistic individuals.

There is a growing body of literature on the role of extended family members in general childcare. Globally, grandparents are key resources in caregiving (Chan et al., 2023; Hayslip et al., 2019), and they are often the first line of support when families encounter challenges (Furstenberg, 2020). However, beyond the role of grandparents, there is limited literature on the roles of other extended family relatives. A review of African American single-mother families suggested adult extended relatives provide a further support network to ensure children’s health and well-being (Jones et al., 2007). Aunts and uncles may mentor other family members or mediate disputes (Milardo, 2010). Some studies also suggested extended relatives provide informal support to children and their parents in settings with limited healthcare resources (Clark et al., 2018; Treleaven, 2023). This support includes assistance with childcare (Clark et al., 2018) as well as financial contributions or decision-making authority held by extended relatives that facilitate children’s access to healthcare (Treleaven, 2023).

In contrast to the general caregiving literature where a few studies have been conducted on other extended relatives, studies identified in the disability literature primarily focus on non-custodial grandparents, suggesting both positive and negative aspects of this role (Hillman, 2007; Lee & Gardner, 2010; Mitchell, 2007; Novak-Pavlic et al., 2022). For example, whereas some grandparents provided emotional, financial, and instrumental support to help families of disabled children (Lee & Gardner, 2010; Mitchell, 2007; Novak-Pavlic et al., 2022), others increased parents’ stress levels, particularly when they were unable to understand the disabilities (Lee & Gardner, 2010), accept the disabled children (Mitchell, 2007), or agree on an intervention approach (Hillman, 2007). Moreover, in addition to providing the forms of support mentioned above, custodial grandparents have reported challenges and specific needs related to raising grandchildren with disabilities as primary caregivers (Hayslip & Kaminski, 2005; Hillman & Anderson, 2019).

While previous reviews suggested that some grandparents contribute to increased stress levels for parents, they did not consider parents’ experiences of being stigmatised by grandparents (Hillman, 2007; Lee & Gardner, 2010; Mitchell, 2007; Novak-Pavlic et al., 2022), a common occurrence among families with autistic children (Ooi et al., 2016). Moreover, although previous reviews acknowledged the role of culture, they highlighted a gap in available evidence regarding how culture influences the involvement of extended relatives (Hillman, 2007; Lee & Gardner, 2010). Among reviews conducted to date, only one rapid review focussed specifically on autism (Hillman, 2007); the others were centred on broader categories of disabilities. This rapid review helpfully reported on grandparents’ experience and influences of grandparents’ role on parents of autistic children. However, the nature of the rapid review precluded a full synthesis of study findings and did not consider the role of other extended relatives, such as autistic children’ s aunts and uncles, or how the role of grandparents impacts the lives of autistic children. More recently, there has been a substantial increase in the number of qualitative studies investigating the role of extended relatives in caring for autistic children (Novak-Pavlic et al., 2022) that is yet to be consolidated. A deeper exploration of the various roles of extended relatives, as well as the cultural patterns linked to the provision of support, is needed.

Therefore, this review aims to explore the perspectives of autistic individuals, parents, and extended family members to gain a comprehensive and widely applicable understanding of this role. As cultures differ with respect to who provides care and how family members interact, this review also explores what cultural factors may influence the role of extended family members to guide the provision of support more effectively for families with autistic individuals. Understanding these cultural dynamics is essential for developing support that aligns with the expectations and lived experiences of families with autistic individuals. Without this cultural consideration, services risk overlooking key caregivers or failing to meet family needs. The review focuses specifically on autism due to the unique care demands associated with caring for autistic children (Hayes & Watson, 2013). The specific objectives of this review are to

1. Identify qualitative studies on the role of extended family members within the families, in supporting autistic individuals and their parents and appraise the quality of the identified studies.
2. Synthesise the literature on the role extended family members play in the care of autistic individuals.
3. Synthesise the factors influencing the involvement of extended family members in supporting autistic individuals and their parents.
4. Provide suggestions for future research and priorities for intervention development and clinical practice to equip extended family members with the skills and knowledge needed to provide support to their families.

## Method

### Protocol Registration and PRISMA Guidelines

We followed the updated Preferred Reporting Items for Systematic Reviews and Meta-Analyses (PRISMA) statement guidelines. A protocol was registered with PROSPERO in April 2022 (CRD42022327223), and the qualitative evidence synthesis was conducted following the Enhancing Transparency in Reporting the Synthesis of Qualitative Research (ENTREQ) statement (Tong et al., 2012).

### Search Strategies

We searched PsycINFO, Global Health, MEDLINE, Embase, Web of Science Core Collection, CINAHL, Global Index Medicus, and African Journals Online (AJOL) in April 2022, and then updated the search in September 2023 and March 2025. No language, location or time restrictions were imposed. The Sample, Phenomenon of Interest, Design, Evaluation, Research type (SPIDER) tool was adapted and used to guide the search strategy (Cooke et al., 2012). Following the adaptation by Tuck et al. (2023), we combined Design and Research Type using “or”. Search terms were categorized into “autism” (Sample), “extended family members” (Phenomenon of Interest), “experience and perspectives” (Evaluation), and “qualitative” (Design or Research type). All the categories were combined with Boolean operator “and”. The reference list of selected articles and articles citing the included studies were searched to identify studies missed in the database search. Full search terms are reported in Supplementary Material A.

### Selections of Studies and Inclusion Criteria

**Fig. 1:**
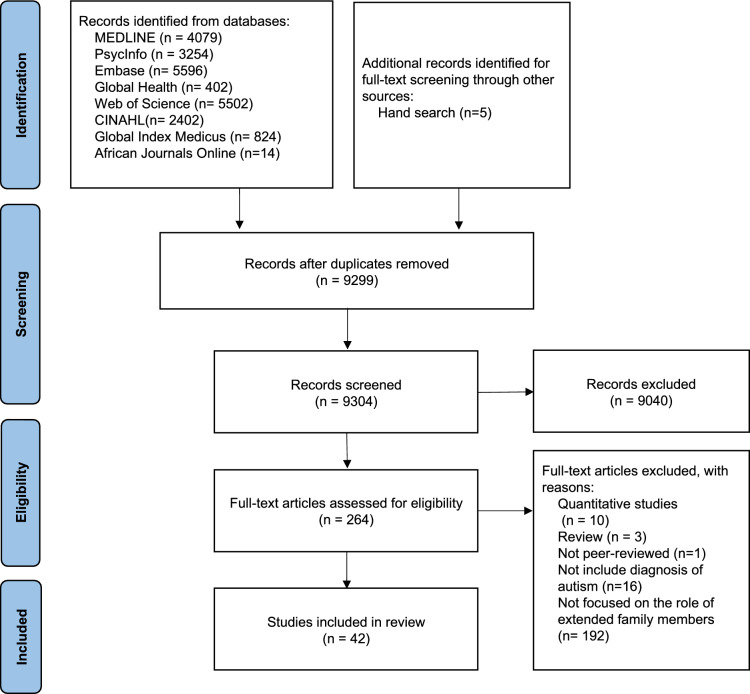
PRISMA flow diagram (Moher et al., 2009) of the study selection process

Figure 1 outlines the search process in accordance with the Preferred Reporting Items for Systematic Reviews and Meta-Analyses (PRISMA) guidelines (Moher et al., 2009). Author JLL ran preliminary searches in the specified databases and downloaded titles and abstracts into EndNote for deduplication. After removing the duplicates, titles and abstracts of the remaining studies were put into the Rayyan for double screening (Ouzzani et al., 2016). Authors JLL and THK each independently reviewed the titles and abstract of studies and excluded the articles that did not meet the inclusion criteria. Both authors then read the full text of selected studies to confirm inclusion or exclusion against the criteria.

Primary research studies using qualitative methods to explore the perspectives of the role of extended family members within the families of autistic individuals were sought. We focussed on synthesising qualitative studies as this allows for an in-depth, nuanced understanding of participants’ lived experiences and helps identify themes across different countries and stakeholder groups (Thomas & Harden, 2008). This level of detail is often not captured in quantitative research. We included the studies focussed on autistic individuals of all ages, and studies centred around a wider range of developmental disabilities with at least some of individuals having a diagnosis of autism.

To incorporate perspectives from various stakeholders, we included studies involving autistic individuals, parents, or extended family members as participants. In particular, we considered studies where either autistic individuals or their parents discussed the role of extended family members, or where extended relatives shared their own experiences. We excluded the studies focussed on the role of siblings, as these are nuclear family members. Full inclusion and exclusion criteria are reported in Supplementary Material B.

### Quality Appraisal

JLL assessed the quality of selected studies using the Critical Appraisal Skills Programme (CASP) checklist for qualitative study (available from https://casp-uk.net). To ensure consistency in quality assessment, THK and RAH assessed the quality of 20% of the included articles, and JLL independently evaluated the same set. Any disagreements were resolved through discussion. Following this, JLL applied the agreed-upon assessment scheme to evaluate the remaining articles. Each item on the CASP checklist was assigned a numerical outcome (No = 0, Can’t tell = 0.5, Yes = 1), resulting in a maximum total score of 10 (Butler et al., 2020). The total CASP score for all papers was used to categorise the methodological quality as either “high” (> 8), “moderate” (6–8), or “low” (≤ 5). Quality appraisal was not used to exclude studies, but rather to evaluate the presented results and facilitate comparisons across studies.

### Data Extraction

For each study, JLL extracted information on authors, publication year, country of study, aims, participants, disabilities considered, age range of autistic individuals, methodology, recruitment, method of data collection, and method of data analysis. The Results and Discussion sections of each study were extracted and uploaded into the qualitative data analysis software NVivo-12 for analysis.

### Synthesis

Thematic synthesis was applied (Thomas & Harden, 2008). This method is suitable for synthesizing the findings from multiple qualitative studies. We used template thematic analysis to develop analytical themes (Brooks et al., 2015). Our template was generated based on existing theories and conceptual frameworks. The family support framework for families of children with disabilities (Kyzar et al., 2012) informed the template of support from extended family members. Similarly, a framework of stigma associated with mental health (Thornicroft et al., 2022) shaped the template of unhelpful or lack of support from extended family members. Family system theory (Turnbull et al., 2010) and the circumplex model (Olson et al., 2019) guided the template of potential factors that may influence the role of extended family members. Full descriptions of each theory and framework are reported in Supplementary Material C.

The three main stages of thematic synthesis were followed: line by line coding, development of descriptive themes based on initial codes, and generation of new analytical themes (Thomas & Harden, 2008). JLL extracted all relevant text, including participants quotations and authors’ thematic interpretations, from the results sections of the selected studies. Relevant expanded analysis and reflections regarding culture related to extended relatives and caregiving expectations were also extracted from the included manuscripts’ discussion sections. Then, JLL familiarised herself with the selected text and proceeded to code the selected text line by line. These codes were then continually revised and reflexively interpreted throughout the process. During the first and second stages, the results and discussion pertaining to the role of extended family members were coded, and then similarities between the codes were identified to develop descriptive themes that capture the meaning of groups of initial codes. In the first stage, coding was mostly inductive; in the second stages, existing theories and conceptual frameworks were used to more deductively organise the initial codes into descriptive themes.

In the third stage, analytical themes were generated based on the examination of the descriptive themes. This stage aimed to go beyond the primary reported data by re-interpreting and synthesizing the findings across studies to generate additional understanding in relation to the role of extended family members. It also allowed for the generation of themes not fully captured by the template. Themes were developed iteratively in this stage. Initial theme development was done by JLL and shared with selected illustrative quotes with the wider author team (MWN and RAH). Themes and subthemes were then refined based on the discussions, allowing for investigator triangulation to increase the robustness of the analyses (Flick et al., 2004).

## Results

### Characteristics of Included Studies

Our searches of database and other sources yielded 22,078 results. After removing duplicates, 9304 records remained for title and abstract screening, from which we assessed 264 full-text articles for eligibility (see Fig. 1). A total of 42 studies, including 40 qualitative and 2 mixed-methods studies, were identified for inclusion in the current review as summarized in Table 1. Most studies focussed on children: 27 studies were on children under 18 years, 11 studies included both children and adults, 1 study included only adults, and 3 did not specify the age range. Among the studies that included autistic adults, the reported age range was 18 to 44 years. Studies were conducted in the US (*n* = 16), China/Taiwan (*n* = 6), Australia (*n* = 3), Canada (*n* = 3), Ghana (*n* = 2), the UK (*n* = 1), Ireland (*n* = 1), New Zealand (*n* = 1), Sweden (*n* = 1), Spain (*n* = 1), South Korea (*n* = 1), Indonesia (*n* = 1), United Arab Emirates (*n* = 1), Ethiopia (*n* = 1), Palestine (*n* = 1), Turkey (*n* = 1), and Brazil (*n* = 1), between 1997 and 2025. Parents’ views were explored in 32 studies, while grandparents’ experiences were considered in 14 publications (with some studies including perspectives from multiple respondent types). Most studies were written in English, except for one in French and one in Chinese. Data from the Chinese study were translated and extracted by JLL who is fluent in Chinese, while the French study was translated and then checked by a colleague fluent in French.

**Table 1:** Characteristics of selected studies

	Author	Country	Participants	Autism and a wider range of disabilities	Age of autistic individual/s	Methodology	Recruitment	Data collection	Data analysis	Aim/s
1	Ben-Cheikh and Rousseau (2013)	Canada	10 Immigrant north African Parents	All children were on the autism spectrum	2 to 6 years	Qualitative	Purposive sampling	Semi-structured interviews & participant observation	Content analysis	To explore the impact of having an autistic child on the social support networks of parents of North African origin who are new immigrants to Quebec
2	Blanche et al. (2015)	US	15 Parents	All children were on the autism spectrum	3 to 8 years	Qualitative	Purposive & Snowball sampling	Semi-structured interviews	Thematic analysis	To understand the caregiving experiences of Latino families with children with ASD, including daily activities, coping strategies, and service utilization
3	Bobadilla (2021)	US	6 Hispanic fathers	All children were on the autism spectrum	N/A	Qualitative	Convenience sampling	Semi-structured interviews	Interpretive phenomenological analysis	To understand the impact and experiences of ASD on Hispanic families and their conceptualization of fatherhood
4	Casillas et al. (2017)	US	5 Latino and 6 non-Latino White parents	All children were on the autism spectrum	2.5 to 13 years	Qualitative	Purposive sampling	Semi-structured interviews	Grounded theory	To understand the cultural differences in the experiences of Latino and non-Latino White parents who are raising an autistic child
5	Çetin et al. (2020)	Turkey	10 Parents	All children were on the autism spectrum	5 to 23 years	Qualitative	Purposive sampling	In-depth interviews	Thematic content analysis	To evaluate the perceptions of social stigma among parents of children having autism and their ways of coping with stigma
6	Coleman et al. (2023)	US	9 Parents	All children were on the autism spectrum	3 to 18 years	Qualitative	Convenience sampling	Semi-structured interviews	Consensual qualitative research (CQR)	To explore the perceived familial support of parents of children with autism spectrum disorder (ASD) to better understand the assistance and gaps they experienced
7	D'Astous et al. (2013)	US	14 Grandparents	All grandchildren were on the autism spectrum	N/A	Qualitative	Convenience sampling	Interviews	Framework analysis	To discover the range of engagement within intergenerational relationships in families with a child with an ASD
8	Dababnah and Parish (2013)	Palestine	24 Palestinian Arab parents	All children were on the autism spectrum	4 to 17 years	Qualitative	Purposive	Interviews & Focus groups	Grounded theory	To examine parents’ knowledge, attitudes, burdens, and coping strategies related to caring for an autistic child in the West Bank
9	DuBay et al. (2018)	US	20 Latino parents	All children were on the autism spectrum	1 to 6 years	Mixed methods	Purposive sampling	Focus groups	Thematic analysis	To explore how culturally appropriate, feasible, and acceptable Latino caregivers perceived intervention models, strategies, and targets
10	Fauziah et al. (2021)	Indonesia	20 Parents (10 couples)	All children were on the autism spectrum	5 to 17 years	Mixed methods	Purposive & convenience sampling	Survey & Semi-structured interview	Thematic analysis	To investigate the dynamics of family harmony in families of autistic children
11	Hillman and Anderson (2019)	US	117 Custodial grandparents	All children were on the autism spectrum	2 to 44 years	Qualitative	Convenience sampling	Open-ended survey questions	Grounded theory analysis	To examine perspectives of custodial grandparents of children with ASD, including their sources of both stress and to generate recommendations regarding how best to support these caregivers
12	Hillman et al. (2017)	US	1870 non-custodial grandparents	All children were on the autism spectrum	1 to 42 years	Qualitative	Convenience sampling	Open-ended survey questions	Grounded theory analysis	To understand experiences of non-custodial grandparents of autistic children, including both positive and negative aspects of grandparenting
13	Huang et al. (2011)	China/Taiwan	16 Chinese fathers	1 autistic child, and 15 children with other DD	1 to 10 years	Qualitative	Purposive	In-depth interviews	Hermeneutic phenomenology	To explore fathers’ experiences of having their child diagnosed with a developmental disability in the context of Chinese culture
14	Huang et al. (2020)	China/Taiwan	25 Grandmothers	At least 1 autistic grandchild	7 months to 9 years	Qualitative	Purposive sampling	Semi-structured interviews	Phenomenological analysis	To explore the lived experience of grandmothers caring for a grandchild with a developmental delay or disability in the context of Chinese culture
15	Huang et al.(2023)	China	14 Mothers	All children were on the autism spectrum	3 to 15 years	Qualitative	Purposive and snowball sampling	In-depth interviews	Thematic narrative analysis	To examine the experiences of mothers of autistic children in navigating between caregiving and working life in China
16	Lamba et al. (2022)	United Arab Emirates	17 Expat mothers	All children were on the autism spectrum	5 to 22 years	Qualitative	Purposive and snowball sampling	In-depth and semi-structured interviews	Thematic analysis	To explore challenges and support structures of mothers with children with ASD in the UAE
17	LaRoche, & des Rivières-Pigeon, (2022)	Canada	17 parents & 1 grandmother	All children were on the autism spectrum	4 to 10 years	Qualitative	Purposive sampling	Semi-structured interviews	Thematic analysis	To examine the social support of caregivers of children with ASD living in Québec, Canada
18	Lee and Gardner (2015)	South Korea	6 Mothers	3 autistic children and 3 children with other DD	5 to 11 years	Qualitative	Purposive sampling	Semi-structured interviews	Phenomenological analysis	To explore South Korean mothers’ perceptions of grandparents’ roles and support for families of children with disabilities
19	Lilley et al. (2020)	Australia	11 Mothers and 1 grandmother; Aboriginal and Torres Strait Islander	All children were on the autism spectrum	2 to 22 years	Qualitative	Purposive sampling	Semi-structured interviews	Reflexive thematic analysis	To explore the experiences of Aboriginal and Torres Strait Islander women supporting their autistic children
20	Lopez et al. (2018)	US	44 Latina mothers and 52 White mothers	All children were on the autism spectrum	2 to 22 years	Qualitative	Purposive sampling	Interviews and open-ended survey questions	Thematic analysis	To identify similarities and differences between White and Latino families with respect to their reaction to the diagnosis and what kind of support extended family members provide to parents
21	Lu et al. (2022)	China	12 Grandparents	4 Autistic grandchildren and 8 grandchildren with other DD	2.5 to 5 years	Qualitative	Purposive sampling	Semi-structured interviews	Phenomenological analysis	To explore the posttraumatic growth experience of grandparents of children with developmental disabilities under 6 years old
22	Margetts et al. (2006)	UK	6 Grandparents	All children were on the autism spectrum	3 to 5 years	Qualitative	Purposive sampling	Semi-structured interviews	General inductive method	To explore the experience of being a grandparent of an autistic grandchild
23	Mbamba et al. (2023)	Ghana	15 Mothers	All children were on the autism spectrum	1 to 12 years	Qualitative	Convenience sampling	In-depth interviews	Thematic analysis	To investigate the lived experiences of single mothers caring for their autistic children in Ghana and identify the support systems available to improving the welfare of their children
24	Miller et al. (2012)	Australia	22 Grandparents	4 autistic grandchildren and 18 grandchildren with other disabilities	2 to 14 years	Qualitative	Purposive sampling	Semi-structured interviews	Thematic analysis	To explore grandparents’ experiences of caring for a child with a disability and the impact on their family relationships and quality of life
25	Mirfin-Veitch et al.(1997)	New Zealand	12 Parent-Grandparent pairs	At least 1 autistic grandchild	5 to 15 years	Qualitative	Purposive sampling	In-depth interviews	General inductive method	To explore factors and characteristics related to the extent to which support is a component of parent–grandparent relationships in families of children with disabilities
26	Myers et al. (2009)	US	493 Parents; 77% in the US	All children were on the autism spectrum	75% children aged 3–11; 3 children were younger than 2; 12 children aged 18–21	Qualitative	Convenience sampling	Open-ended survey question	Content analysis	To understand how raising a child with autism affected parental lives and the lives of their families
27	Neely-Barnes et al. (2010)	US	45 Parents	11 autistic children and 34 children with other DD	1 to 21 years	Qualitative	Purposive sampling	Focus groups	Interpretive method	To explore the communication and activities occurring outside of the family or in the community
28	Neely-Barnes et al.(2011)	US	11 Parents	All children were on the autism spectrum	1 to 15 years	Qualitative	Purposive sampling	Focus groups	Interpretive method	To explore public perceptions of autism and parental conceptualizations of themselves and their children
29	Oti-Boadi et al.(2020)	Ghana	6 Mothers	All children were on the autism spectrum	5 to 18 years	Qualitative	Purposive sampling	Semi-structured interviews	Thematic network analysis	To explore the stigma experiences of mothers of children with ASD and the role of forgiveness in helping them adjust and relate well with offending persons
30	Pinto et al. (2016)	Brazil	10 Parents	All children were on the autism spectrum	N/A	Qualitative	Purposive sampling	Semi-structured interviews	Thematic content analysis	To analyse the context in which the diagnosis of autism is revealed and the impact of this revelation on family relationships
31	Prendeville and Kinsella (2019)	Ireland	9 Families, including 12 parents, 12 grandparents	All children were on the autism spectrum	5 to 18 years	Qualitative	Snowball sampling	Semi-structured interviews	Thematic analysis	To explore how grandparents support children with autism and their parents using a family systems perspective
32	Shanmugarajah et al. (2022)	Canada	8 Immigrant Sri Lankan Tamil mothers	All children were on the autism spectrum	16 to 23 years	Qualitative	Purposive sampling	Semi-structured interviews	Content analysis	To better understand the experiences of immigrant Sri Lankan Tamil parents of children diagnosed with ASD in Southern Ontario, Canada
33	Tekola et al. (2020)	Ethiopia	18 Parents	6 autistic children and 8 children with other DD	4 to 9 years	Qualitative	Purposive sampling	In-depth interviews	Thematic analysis	To explore perceptions and experiences of stigma among parents of children with DD in Ethiopia and examine the contributing and protective factors for internalised stigma based on the perspectives of the parents themselves
34	Woodbridge et al. (2011)	Australia	22 Grandparents	4 autistic grandchildren and 18 grandchildren with other disabilities	2 to 14 years	Qualitative	Purposive sampling	Semi-structured interviews	Thematic analysis	To explore how having a grandchild with a disability influences grandparents' sense of identity and enactment of the grandparent role
35	Yang et al. (2018)	US	9 White Grandparents	6 grandchildren with autism, and 2 with other DD;	6 to 17 years	Qualitative	Purposive	Semi-structured interviews	N/A	To examine the roles and experiences of grandparents supporting children with disabilities
36	Zakirova-Engstrand et al.(2020)	Sweden	17 Parents	All children were on the autism spectrum	2–6 years	Qualitative	Purposive sampling	Semi-structured interviews	Content analysis	To investigate explanatory models of autism among parents of young children with ASD in the multicultural context of Sweden
37	Zechella & Raval (2016)	US	15 Asian Indian parents (7 couples and 1 mother)	4 autistic children and 4 children with other DD	6 to 23 years	Qualitative	Purposive & Snowball sampling	Semi-structured interviews	Thematic analysis	To examine unique challenges experienced by Asian Indian parents of children with IDD in US focussing on the cultural explanations of disability, sources of stress and support, immigration experience, and perceptions of the child’s future
38	Sanderson and Aquino (2023)	US	23 parents (Asian; Black or African American; Hispanic or Latinx; White)	10 autistic adults and 13 with other developmental disabilities	18 to 29 years	Qualitative	Purposive & convenience sampling	Semi-structured interviews	Grounded theory analysis	To explore the type of natural supports parents of adults with DD provide, identified other members of natural support networks, and challenges families face in securing and maintaining natural supports
39	Wang et al.(2023)	China	12 Parents	All children were on the autism spectrum	3 to 8 years	Qualitative	Purposive & snowball sampling	in-depth interviews	Content analysis	To investigate the parenting stress and coping experiences of Chinese parents raising children with autism from a cultural standpoint
40	Klitzman et al. (2025)	US	28 Parents (12 are couples from one family)	All children were on the autism spectrum	4 to 30 years	Qualitative	Purposive sampling	Semi-structured interview	Grounded theory analysis	To assess whether genetic test results identifying the cause of a child’s autism alter how parents perceive and treat their child
41	Bai et al. (2024)	China	21 Parents	All children were on the autism spectrum	2 to 6 years	Qualitative	Purposive sampling	Semi-structured interview	Thematic analysis	To explore the factors that parents perceive as influencing their adjustment to caring for a child with autism
42	Baena et al. (2024)	Spain	17 Grandparents	All grandchildren were on the autism spectrum	2 to 15 years	Qualitative	Convenience & snowball sampling	Semi-structured interview	Thematic analysis	To explore the experiences of grandparents of children on the autism spectrum in the Spanish context

Although only countries are mentioned here, many articles involved a variety of ethnicities or immigrant populations in their participant samples: 5 studies from the US focussed on ethnic/immigrant groups (2 comparative studies between white and Latinx parents, 2 studies focussed fully on Latinx parents, and 1 paper focussed fully on Indian parents); 2 studies in Canada considered, respectively, South Asian immigrants and African immigrants; 1 study in Australia focussed fully on Aboriginal and Torres Strait Islander communities.

### Methodological Quality of Included Studies

The methodological quality of all the studies included in the analysis was assessed and categorized as either high (*n* = 37) or moderate (*n* = 5). Detailed quality assessment ratings are shown in Table 2. Generally, the studies rigorously reported their analyses and provided detailed procedures. A notable limitation was noted in the lack of reflection on the relationship between researchers and participants in 12 studies. As there is no empirically tested method for excluding qualitative studies from synthesis based on quality (Thomas & Harden, 2008), no studies were excluded.

**Table 2:** Quality of included studies

Authors and publication year	1. Was there a clear statement of the aims of the research?	2. Is a qualitative methodology appropriate?	3. Was the research design appropriate to address the aims of the research?	4. Was the recruitment strategy appropriate to the aims of the research?	5. Was the data collected in a way that addressed the research issue?	6. Has the relationship between researcher and participants been adequately considered?	7. Have ethical issues been taken into consideration?	8. Was the data analysis sufficiently rigorous?	9. Is there a clear statement of findings?	10. Is the research valuable?	Quality judgement
Ben-Cheikh and Rousseau (2013)	Yes	Yes	Yes	Yes	Yes	No	Can’t tell	Can’t tell	Yes	Yes	Moderate (8)
Blanche et al. (2015)	Yes	Yes	Yes	Yes	Yes	Yes	Yes	Yes	Yes	Yes	High (10)
Bobadilla (2021)	Yes	Yes	Yes	Yes	Yes	Yes	Yes	Yes	Yes	Yes	High (10)
Casillas et al. (2017)	Yes	Yes	Yes	Yes	Yes	Yes	Yes	Yes	Yes	Yes	High (10)
Çetin et al. (2020)	Yes	Yes	Yes	Can’t tell	Yes	Can’t tell	Yes	Yes	Yes	Yes	High (9)
Coleman et al. (2023)	Yes	Yes	Yes	Yes	Yes	Yes	Yes	Yes	Yes	Yes	High (10)
D’Astous et al. (2013)	Yes	Yes	Yes	No	Yes	No	Yes	No	Yes	Yes	Moderate (7)
Dababnah and Parish (2013)	Yes	Yes	Yes	Yes	Yes	Yes	Yes	Yes	Yes	Yes	High (10)
DuBay et al. (2018)	Yes	Yes	Yes	Yes	Yes	Yes	Yes	Yes	Yes	Yes	High (10)
Fauziah et al. (2021)	Yes	Yes	Yes	Yes	Yes	No	Can’t tell	Can’t tell	Yes	Yes	Moderate (8)
Hillman and Anderson (2019)	Yes	Yes	Can’t tell	Yes	Yes	No	Yes	Yes	Yes	Yes	High (8.5)
Hillman et al. (2017)	Yes	Yes	Can’t tell	Yes	Yes	No	Yes	Yes	Yes	Yes	High (8.5)
Huang et al. (2011)	Yes	Yes	Yes	Yes	Yes	Yes	Yes	Yes	Yes	Yes	High (10)
Huang et al. (2020)	Yes	Yes	Yes	Yes	Yes	Yes	Yes	Yes	Yes	Yes	High (10)
Huang et al. (2023)	Yes	Yes	Yes	Yes	Yes	Yes	Yes	Yes	Yes	Yes	High (10)
Lamba et al. (2022)	Yes	Yes	Yes	Yes	Yes	Yes	Yes	Yes	Yes	Yes	High (10)
LaRoche & des Rivières-Pigeon (2022)	Yes	Yes	Yes	Yes	Yes	No	Yes	Can’t tell	Yes	Yes	High (8.5)
Lee and Gardner (2015)	Yes	Yes	Yes	Yes	Yes	Yes	Yes	Yes	Yes	Yes	High (10)
Lilley et al. (2020)	Yes	Yes	Yes	Yes	Yes	Yes	Yes	Yes	Yes	Yes	High (10)
Lopez et al. (2018)	Yes	Yes	Yes	Yes	Yes	Yes	Yes	Yes	Yes	Yes	High (10)
Lu et al. (2022)	Yes	Yes	Yes	Yes	Yes	No	Yes	Yes	Yes	Yes	High (9)
Margetts et al. (2006)	Yes	Yes	Yes	Yes	Yes	Can’t tell	Yes	Yes	Yes	Yes	High (9.5)
Mbamba et al. (2023)	Yes	Yes	Yes	Yes	Yes	Yes	Yes	Yes	Yes	Yes	High (10)
Miller et al. (2012)	Yes	Yes	Yes	Yes	Yes	Yes	Yes	Yes	Yes	Yes	High (10)
Mirfin-Veitch et al.(1997)	Yes	Yes	Yes	Yes	Yes	No	Can’t tell	Can’t tell	Yes	Yes	Moderate (8)
Myers et al. (2009)	Yes	Yes	Can’t tell	Yes	Can’t tell	No	Yes	Yes	Yes	Yes	Moderate (8)
Neely-Barnes et al. (2010)	Yes	Yes	Yes	Yes	Yes	Yes	Yes	Yes	Yes	Yes	High (10)
Neely-Barnes et al.(2011)	Yes	Yes	Yes	Yes	Yes	Yes	Yes	Yes	Yes	Yes	High (10)
Oti-Boadi et al.(2020)	Yes	Yes	Yes	Yes	Yes	Yes	Yes	Yes	Yes	Yes	High (10)
Pinto et al. (2016)	Yes	Yes	Yes	Yes	Yes	No	Yes	Can’t tell	Yes	Yes	High (8.5)
Prendeville and Kinsella (2019)	Yes	Yes	Yes	Yes	Yes	Yes	Yes	Yes	Yes	Yes	High (10)
Shanmugarajah et al. (2022)	Yes	Yes	Yes	Yes	Yes	Can’t tell	Yes	Yes	Yes	Yes	High (9.5)
Tekola et al. (2020)	Yes	Yes	Yes	Yes	Yes	Yes	Yes	Yes	Yes	Yes	High (10)
Woodbridge et al. (2011)	Yes	Yes	Yes	Yes	Yes	Yes	Yes	Yes	Yes	Yes	High (10)
Yang et al. (2018)	Yes	Yes	Yes	Yes	Yes	Yes	Yes	Yes	Yes	Yes	High (10)
Zakirova-Engstrand et al.(2020)	Yes	Yes	Yes	Yes	Yes	Yes	Yes	Yes	Yes	Yes	High (10)
Zechella & Raval (2016)	Yes	Yes	Yes	Yes	Yes	Yes	Yes	Yes	Yes	Yes	High (10)
Sanderson and Aquino (2023)	Yes	Yes	Yes	Yes	Yes	Can’t tell	Yes	Yes	Yes	Yes	High (9.5)
Wang et al.(2023)	Yes	Yes	Yes	Yes	Yes	No	Yes	Yes	Yes	Yes	High (9)
Klitzman et al. (2025)	Yes	Yes	Yes	Yes	Yes	No	Yes	Yes	Yes	Yes	High (9)
Bai et al. (2024)	Yes	Yes	Yes	Yes	Yes	Can’t tell	Yes	Can’t tell	Yes	Yes	High (9)
Baena et al. (2024)	Yes	Yes	Yes	Yes	Yes	Can’t tell	Yes	Yes	Yes	Yes	High (9.5)

### Thematic Synthesis

**Fig. 2:**
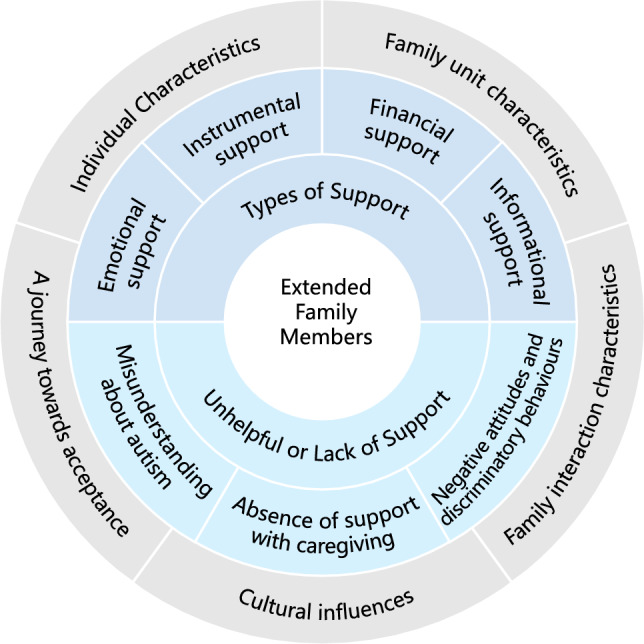
Diagram depicting themes and subthemes in the thematic synthesis

The codes developed through the thematic analysis of the Results and Discussion sections of the selected studies mostly fitted in with the framework. Three themes were developed representing positive and negative aspects of the role of extended family members: (1) *Types of support*; (2) *Unhelpful or Lack of support*; (3) *Factors influencing the role of extended family members.* Figure 2 presents the Diagram depicting themes and subthemes in the thematic synthesis.

### Theme 1 Types of Support

In 39 of the studies, extended family members were highly involved and provided various kinds of support to autistic individuals and their parents. These could be categorised into four types: emotional support, instrumental support, financial support, and informational support.

### Subtheme 1.1 Emotional Support

Parents reported that their extended family members helped to reduce stress and improve emotional well-being (Lopez et al., 2018; Lu et al., 2022). The emotional support received by parents from extended family members included encouragement (Fauziah et al., 2021), prayer (Shanmugarajah et al., 2022), someone to talk to about issues (Casillas et al., 2017), and someone who could empathise with their needs and parenting efforts (Margetts et al., 2006). The provision of emotional support offered parents a sense of relief that they did not have to face the difficulties of caring alone. One parent described their extended family members as being their “rock” and another said they took “the weight off their shoulders” (Coleman et al., 2023).

*“My mother was so good, when I was having very hard nights I would ring her and she would say bring him up… So I’ve been very grateful to my Mum, she has been sick her whole life and she shouldn’t be here at all, she has been and unbelievable amount of strength and support to me she is the main person.” (Parent; *Prendeville & Kinsella, 2019*, p. 742)*

Besides providing emotional support to parents, extended family members supported autistic individuals themselves by showing their unconditional love and acceptance, and by encouraging them (Lee & Gardner, 2015; Yang et al., 2018). With extended family members, acceptance of autistic individuals meant involving them in family activities or seeing them as “*just one of the gang, just one of the family*”(Neely-Barnes et al., 2010, p.8). Parents described when they perceived their children as accepted by other family members, they did not feel embarrassed or ashamed and no longer hid away from others (Tekola et al., 2020).

Grandparents remarked that they celebrated each milestone of their autistic grandchildren and were proud of their grandchild’s development (Hillman & Anderson, 2019; Hillman et al., 2017; Woodbridge et al., 2011; Yang et al., 2018).

*“I have no idea… how her ultimate outcome is going to be. I don’t know how far she can go or how fast. All I am doing is watching this beautiful flower that just keeps opening and opening ... She doesn’t hit the milestone at the same point on the timeline as her siblings. But she hits them. ... And she goes on from there towards the next one” (Grandparents; *Yang et al., 2018*, p. 369)*

### Subtheme 1.2 Instrumental Support

Instrumental support featured prominently among four types of support. The most frequently reported source of instrumental support was from grandparents of autistic individuals, including providing childcare (Casillas et al., 2017; Coleman et al., 2023; Lu et al., 2022), helping with transportation to school or therapy (Huang et al., 2020), assisting with homework (Lee & Gardner, 2015), and housework tasks (Mirfin-Veitch et al., 1997; Woodbridge et al., 2011). Some studies also referred to instrumental support provided by other family members such as autistic individuals’ cousins (Lilley et al., 2020; Sanderson & Aquino, 2023; Shanmugarajah et al., 2022) and parents’ siblings (LaRoche & Rivières-Pigeon, 2022; Pinto et al., 2016).

*“I realise how much she has to run him around everywhere for the certain things. She is on the go constantly, which is why we help her with the housework and things like that. I have to take [child with disability] here, I have to take him there, I have to take him to [location] for his other therapy and things like that. It is fairly constant.” (Grandparent; *Woodbridge et al., 2011*, p. 359)*

Support from grandparents sometimes went beyond the traditional role of grandparents. Grandparents were actively involved in the intervention and therapy for autistic individuals. They attended specialist appointments (Lu et al., 2022; Woodbridge et al., 2011) and applied intervention techniques to help with the development and education of autistic individuals (Lee & Gardner, 2015; Woodbridge et al., 2011; Yang et al., 2018).

*“I used to hope that I could enjoy leisure time after retirement, but now the most important thing for me is to accompany my grandchild for rehabilitation treatment. My greatest wish is for my grandchild to acquire language abilities and reach a state of typical developmental level, and can go to kindergarten in the future.” (Grandparent; *Lu et al., 2022*, p. 2227)*

The instrumental support from grandparents was perceived as essential, allowing the parents to have some vital time for themselves (Lee & Gardner, 2015; Woodbridge et al., 2011), or enabling parents to work and avoid a loss of income, which was particularly crucial for single mothers (Mbamba et al., 2023).

*“I am still working as a government official after the birth of my child with a disability. My mother-in law lives close to me, so she has been raising my child ever since he was born. It would not be possible to be a working mother, who is successful in the workplace and at home simultaneously, without my mother-in-law’s support.” (Parent; *Lee & Gardner, 2015*, p. 214)*

### Subtheme 1.3 Financial Support

Parents reported that their relatives provided financial support in caring for their autistic child, including payment for therapy, school fees, and living costs (Fauziah et al., 2021; LaRoche & Rivières-Pigeon, 2022; Lee & Gardner, 2015; Mbamba et al., 2023). Grandparents described they made sacrifices to cover costs associated with their autistic grandchild’s needs, including special diet expenses and support activities (Hillman & Anderson, 2019). Financial support from extended family members was especially an important source of support for single mothers (Mbamba et al., 2023).

*“We were basically trying to help Olivia and keep Abby afloat. ... I don’t think she could have survived without our financial help because the limits were so low that a family couldn’t exist in order to get health insurance for her children” (Grandparent; *Yang et al., 2018*, p. 365)*

*“My younger sister who is in Germany sends me money periodically to buy things for my child. My sister knows about the running away of my husband when I gave birth to this child, so she pities and helps me” (Parent; *Mbamba et al., 2023*, p. 51)*

### Subtheme 1.4 Informational Support

Parents described that extended family members sometimes were the first to express concerns that their child might have autism which led them to seek formal help (Blanche et al., 2015; Prendeville & Kinsella, 2019; Zakirova-Engstrand et al., 2020). This was especially common if the extended family member had more specialist knowledge of medicine, psychology, or special education (Bobadilla, 2021; Neely-Barnes et al., 2010; Woodbridge et al., 2011). *“I didn’t notice anything. I noticed when he was 1.5 years old after my mother said to me, ‘You need to check him’.” (Parent; *Zakirova-Engstrand et al., 2020*, p. 13).*

*“He was diagnosed I think, when he was two, or two and a half and that’s when my brother-in-law who is studying Psychology, noticed that he was going off to the side, being antisocial. And he also noticed that he wouldn’t see him in the eyes. And he just out of the blue, told my wife, ‘Hey you might want to get him an evaluation, because that doesn’t seem right’.” (Parent; *Bobadilla, 2021*, p. 10)*

Several parents noted that they sought advice from their extended family members, including autistic family members, upon noticing a language delay or behavioural problems in their child (Coleman et al., 2023; Ben-Cheikh & Rousseau, 2013), or sought guidance on accessing services (Lilley et al., 2020). A mother asked questions about her child to autistic relatives like, *“OK he’s doing this. What is he feeling? Why is this happening? How would I help you in the same situation? What would you do? What would help you?” (Parent; Coleman *et al*., *2023*, p. 10).*

### Theme 2 Unhelpful or Lack of Support

This theme covers negative or neutral experiences relating to extended family members (identified in 35 studies), including misunderstanding about autism, absence of support with caregiving, negative attitudes, and discriminatory behaviours.

### Subtheme 2.1 Misunderstanding About Autism

This subtheme captures experiences associated with misunderstandings between parents and extended family members. Parents conveyed that extended family members lacked understanding regarding the cause of autism. This included beliefs such as considering the diagnosis temporary (Blanche et al., 2015), viewing the child as spoiled and in need of more discipline (DuBay et al., 2018; Zakirova-Engstrand et al., 2020), labelling the child as picky (Coleman et al., 2023), and attributing autism to curses, sins, or other religious explanations (Dababnah & Parish, 2013; Oti-Boadi et al., 2020).

*“My family is like very traditional and when my parents and parents-in-law, well my mom is still living so when my parents-in-law, my grandma they all believed it’s like a curse from ancestors; ancestry curse or something. That is the main belief in my side, in Hindu side of the family so we believe that.” (Parent; *Zechella & Raval, 2016*, p. 1299)*

Extended family members also recommended using traditional healing or unscientific methods instead of seeking support services (Lamba et al., 2022; Lopez et al., 2018; Zechella & Raval, 2016). Some extended family members, who did not understand the cause of autism, denied the autism diagnosis (Neely-Barnes et al., 2011) and believed the child would grow out of it (Myers et al., 2009)*.*

Although some extended family members accepted the diagnosis of autism, parents reported misunderstandings in their knowledge of effective behaviour management strategies. One parent explained this by saying,

*“My brother actually paid my son $50 to eat something this summer. It was one bite of each thing. It took him an hour and a half. He gagged, he choked, but he got that $50. I said, ‘You don’t understand. Everyday it’s like that. I’m not paying him money to eat food.’ They don’t get it. They just think if I pressured him enough, if I give him enough grief, he’ll eat the food. They don’t understand that every meal if you push him to eat something, he’ll just throw it up on his plate.” (Parent; *Neely-Barnes et al., 2011*, p. 214)*

Still, some extended family members were reported to be unaware of the requirements for treating autistic individuals (LaRoche & Rivières-Pigeon, 2022). A parent noted that the grandparent did not understand the efforts they put into raising their children, “*Despite our efforts, his grandparents do not seem to understand that we have to spend so much money on his treatments*” (Lee & Gardner, 2015, p. 215).

### Subtheme 2.2 Absence of Support with Caregiving

Parents reported an absence of support with caregiving from their extended family members, commonly due to the living distance (Coleman et al., 2023; D’Astous et al., 2013), and strained family relationship (LaRoche & Rivières-Pigeon, 2022; Lee & Gardner, 2015). In particular, immigrant parents described they struggled to overcome challenges without the support of their extended family members nearby (DuBay et al., 2018; Zakirova-Engstrand et al., 2020). Even when help was available within a short distance, some parents did not receive support because they felt uncomfortable asking for assistance (Lee & Gardner, 2015).

*“I do not feel that I am getting help these days because I am the one who is raising him and we do not live close to my parents’ house. Though my child’s paternal grandparents live close to us, I do not feel as comfortable asking for help as I do with my parents. I believe it would help a lot if my mom lived close to us.” (Parent; *Lee & Gardner, 2015*, p. 215)*

Some parents had other types of support but were not receiving assistance in caring for their children due to the challenges associated with their care. One parent noted that the grandparents provided emotional support; however, they could not assist physically, “*her nerves cannot handle the screaming and crying and she doesn’t change diapers like she says*” (Coleman et al., 2023, p. 10). Moreover, some parents expressed that they did not anticipate receiving additional support with caregiving from extended family members and, instead, relied on themselves (Huang et al., 2023).

*“At the time, I thought, ‘The child is yours. Who can you hand the child to?’ I think many women might think in the same way as I do. Who else can you blame? Who else can you rely on for help? I believe it is already massive support if grandfather and grandmother help me cook. Can you expect them to help you raise the child?” (Parent; *Huang et al., 2023*, p. 12)*

### Subtheme 2.3 Negative Attitudes and Discriminatory Behaviours

This subtheme covers accounts of the negative attitudes and discrimination experienced within the wider family. Stigmatising attitudes and behaviours expressed by extended relatives can be targeted towards the autistic individuals themselves (described in 19 of the studies reviewed) or towards the parents (identified in 24 studies). Since none of the studies reviewed reported on the perspectives of autistic people themselves, all accounts reported here are perspectives from parents.

### Subtheme 2.3.1 Negative Attitudes and Discriminatory Behaviours Against Autistic Individuals

Parents described experiencing difficulties in gaining acceptance for their autistic child from extended family members (Pinto et al., 2016; Tekola et al., 2020; Woodbridge et al., 2011). A mother noted, *“… I thought a lot about the prejudice of people and my husband’s family.” (*Pinto et al., 2016*, p. 5).* Some parents reported the negative stereotypes that extended family members held about their autistic children (Huang et al., 2011; Lilley et al., 2020), for instance, “*My parents think my son with problems is unable to achieve anything or do anything well in the future*” (Huang et al., 2011, p. 243).

Informants also described mistreatment of autistic people by extended relatives in various ways, including isolating the individuals from public and family events (Lee & Gardner, 2015; Tekola et al., 2020), avoiding visits and interactions (Lamba et al., 2022; Woodbridge et al., 2011), and suggesting the parents give up on their child (Dababnah & Parish, 2013; Huang et al., 2011).

*“Some of the people from my family were understanding. Others were not. They were telling us to get rid of him. To get rid of him! [They said], ‘Why are you taking care of him?’ That is how some people think. When he got sick, they kept telling us, ‘Why are you even spending money on him?’.” (Parent; *Dababnah & Parish, 2013*, p.1674)*

Parents also noted that some extended family members blamed or judged their autistic children, for their delayed skills development (Lee & Gardner, 2015), or atypical behaviours (Çetin et al., 2020; Lamba et al., 2022).

*“…his aunt and her… kids, although he loves them are not as understanding so they’re always blaming him if he’s being too tough or you know…” (Parent; *Lamba et al., 2022*, p. 7)*

*“…I witnessed that his uncle's brother was laughing at him, laughing at his childlike behaviours. I felt so sorry for that.” (Parent; *Çetin et al., 2020*, p. 15)*

### Subtheme 2.3.2 Negative Attitudes and Discriminatory Behaviours Against Parents

Parents experienced blame from extended family members for their child’s autism. The commonly attributed blame on parents for causing a child’s autism was a mix of biological and supernatural factors. These factors included the belief that parents were impacted by the supernatural power or curses (Oti-Boadi et al., 2020; Tekola et al., 2020; Zechella & Raval, 2016), mothers did wrong things during pregnancy (Lee & Gardner, 2015; Zechella & Raval, 2016), and a family history of autism on the other side of family (Dababnah & Parish, 2013; Huang et al., 2011).

Many parents also highlighted they were blamed for practising bad parenting. One mother noted comments from relatives: “*She [the parent] used to make the baby sit in front of TV the whole day that’s why his speech is delayed*” (Lamba et al., 2022, p. 7), and another said, “*They blamed me for not educating him*” (Lopez et al., 2018, p. 45). Mothers of autistic children were more likely to be blamed than fathers (Lee & Gardner, 2015; Oti-Boadi et al., 2020; Pinto et al., 2016).

*“My mother-in-law said his disability was caused by me because I had done the wrong things during my pregnancy. The ironic part is that she has never blamed her son for having a child who has a disability, which means she did not make it my husband’s responsibility but mine. Her words brought me sadness and depression.” (Parent; *Lee & Gardner, 2015*, p. 212)*

Parents frequently reported being isolated, excluded, and rejected. Extended family members excluded parents due to their embarrassment of being associated with a child with a disability (Tekola et al., 2020) and a fear of being harmed by the autistic children (Çetin et al., 2020; Oti-Boadi et al., 2020).

*“I am excluded by my family including my mother…Their problem is embarrassment. They told me not to bring my child to their house during daytime [when people can see her] and they told me to bring her to their house during the night-time [so that nobody can see her]. But only Satan moves at dark. We, children of God, will move in the daytime. They are worried about their dignity.” (Parent; *Tekola et al., 2020*, p. 4)*

These negative attitudes and discriminatory behaviours against autistic individuals and parents placed a great pressure on them (Lee & Gardner, 2015). Some parents described withholding their children’s autism from extended family members to prevent worries (Ben-Cheikh & Rousseau, 2013; Shanmugarajah et al., 2022), feelings of shame (Lilley et al., 2020), or because they think extended family members would not be able to understand autism (Lopez et al., 2018). Similarly, to avoid the expected discrimination, parents commonly reported that they isolated themselves and their children from the wider family.

*“I told the diagnosis to some of my relatives. He is a different child, true; whether they accept him as he is or not does not interest me. He is my child and I love him. I did not mind not to see my own family for three years after the diagnosis.” (Parent; *Çetin et al., 2020*, p. 15)*

### Theme 3 Factors Influencing the Role of Extended Family Members

This theme encompasses factors associated with support or lack of support provided by extended family members, including characteristics of individuals within the family, the family unit, or family interaction, as well as cultural influences (identified in 40 studies). The theme also captures the evolving role of extended family members over time, depending on the changing needs of autistic individuals and their family (Yang et al., 2018), and on the changing levels of acceptance of their relative’s autism diagnosis (Pinto et al., 2016).

### Subtheme 3.1 A Journey Towards Acceptance of Autism

Some extended family members had a change in their initial negative attitude towards autistic individuals and began to accept autism after being educated (Lee & Gardner, 2015; Oti-Boadi et al., 2020; Zechella & Raval, 2016). Parents reported spending a substantial amount of time and effort explaining autism and their child’s needs to other family members (LaRoche & Rivières-Pigeon, 2022; Lilley et al., 2020; Neely-Barnes et al., 2010; Zakirova-Engstrand et al., 2020; Zechella & Raval, 2016), included them in training sessions, or provided them with informational resources (LaRoche & Rivières-Pigeon, 2022).

*“My dad, he was the most, my dad and my sister were the people that were most in denial. Now my dad probably wears, or has decals in the truck that says autism awareness. So, and then he’s very religious. So, when his religious group, he always has him in their prayers. He always has him by word of mouth everywhere.” (Parent; *Bobadilla, 2021*, p. 10)*

Some grandparents noticed feeling less stressed and more accepting as they observed the development of their grandchild (Margetts et al., 2006). Grandparents conveyed feelings of guilt over initially denying the diagnosis and suggested the denial must be confronted, even at the risk of straining their relationship with the adult children (Hillman et al., 2017).

*“It’s hardest at the beginning, when things aren’t right, you don’t know why or what will happen.” (Grandparent; *Margetts et al., 2006*, p. 571)*

*“I still feel great shame and sadness that I didn’t recognize [my grandson’s] condition earlier. Clearly, I was in denial. I haven’t forgiven myself for this to this day.” (Grandparent; *Hillman et al., 2017*, p. 2962)*

### Subtheme 3.2 Characteristics of Individual Family Members

This subtheme describes how characteristics of individual family members may influence the role extended relatives can or want to play in the lives of their autistic relative. Factors of consideration comprise characteristics of the autistic person themselves, difficulties and needs associated with the parents of the autistic child, and characteristics associated with the extended relatives.

#### Characteristics of the Autistic Individual

Autistic individuals’ difficulties were frequently cited as a factor negatively influencing the role of extended family members in providing childcare support. These difficulties included issues such as eloping, meltdowns, and difficulties with toilet training, social skills and eating (Hillman, 2007; Hillman & Anderson, 2019). As one grandparent noted, “*When [my grandson] has one of his anxiety meltdowns…there is no way to comfort him*” (Hillman et al., 2017, p. 2961), while another mentioned, “*he is a picky eater on a gluten free diet*” (Hillman & Anderson, 2019, p. 264). Grandparents felt a loss of control and became reluctant to support in childcare when the grandchild’s behaviours were beyond their understanding (Baena et al., 2024; Huang et al., 2020).

*“(...) we didn’t know, I couldn’t do anything, so I told my R (mother of the adolescent on the autism spectrum) that, while the kid was like that, we couldn’t take care of him, which makes me sad... I got really depressed when I saw him hitting himself like that.”(Grandparent; *Baena et al., 2024*, p.7 )*

#### Characteristics of the Parent

Parental mental health problems or personal difficulties were reasons why some relatives take on more responsibility caring for autistic family members (Hillman & Anderson, 2019; Mbamba et al., 2023; Prendeville & Kinsella, 2019). Single mothers described they were relying on any types of support provided by extended family members because they were unable to leave their autistic child unsupervised (Mbamba et al., 2023). In a study exploring the experiences of custodial grandparents, grandparents reported taking on the primary caregiver’s role due to their own child’s difficulties, including depression, divorce, inability to manage the autistic child's behaviours, or poverty (Hillman & Anderson, 2019).

*“She had a nervous breakdown, with her personal problems it is very difficult for her to have a child so severe, she is trying herself to cope, there at times and it’s difficult, and I think we could give her a bit more support.” (Grandparent; *Prendeville & Kinsella, 2019*, p. 743)*

#### Characteristics of the Extended Family Member

Extended family members’ age, gender, knowledge, and attitudes towards autism were noted to impact their involvement in supporting autistic relatives and their parents.

Grandparents expressed concerns that they would no longer be available to provide support for their families as they got older (Miller et al., 2012; Prendeville & Kinsella, 2019), while parents shared similar worries (Prendeville & Kinsella, 2019).

*“I pay more attention to my health now than before. I always pay attention to avoid getting sick so that I can take care of him [grandson].” (Grandparent; *Lu et al., 2022*, p. 2228)*

Parents had different perceptions regarding the support provided by maternal and paternal relatives, and the gender divisions in support (LaRoche & Rivières-Pigeon, 2022; Lee & Gardner, 2015). Maternal grandparents were identified as more actively involved in supporting families than paternal grandparents (D’Astous et al., 2013; Lee & Gardner, 2015; Prendeville & Kinsella, 2019). Similarly, parents reported they received more support with childcare from female relatives (LaRoche & Rivières-Pigeon, 2022; Lopez et al., 2018), especially grandmothers (LaRoche & Rivières-Pigeon, 2022; Lee & Gardner, 2015; Lopez et al., 2018; Mirfin-Veitch et al., 1997).

*“The maternal grandfather has interest in his grandchild, but since he does not take him to school or therapy centers, he does not seem to know how difficult it is to raise and educate a child with a disability.” (Parent; *Lee & Gardner, 2015*, p. 214)*

Extended family members with a greater understanding of autism were found to be more involved in caring for autistic individuals when compared to those with limited knowledge (D’Astous et al., 2013; Tekola et al., 2020). Limited knowledge hindered extended family members’ abilities to help in the care and intervention needed for autistic individuals (Huang et al., 2023). This includes a lack of basic understanding of the nature of autism, the importance of early intervention, realistic expectations regarding the child’s development, and awareness of the emotional and financial challenges faced by parents (D’Astous et al., 2013; Lee & Gardner, 2015; Klitzman et al., 2025). Notably, extended relatives who received and possessed accurate information about autism adapted better and tended to have a more positive attitude towards it (D’Astous et al., 2013; Klitzman et al., 2025). They believed their support will help their autistic relatives achieve their developmental potential, motivating them to stay involved (Huang et al., 2020). This involvement was not hindered by focussing on their own feelings or questioning their circumstances (Lu et al., 2022; Miller et al., 2012).

*“My mother was hoping that my daughter would outgrow it, and that we’d just misinterpreted her results. She wants to see my daughter live independently and do everything all of the rest of the grandkids do. My mother definitely adjusted when she saw the genetic results, and accepted, ‘It is what it is, and she’ll be as good as we can help her be.’” (Parent; Klitzman et al., *2025*, p. 291)*

*“You basically think to yourself, well what can I do to contribute to him? Well you know they can’t fully recover, but you have to think about how you can approach the situation so that you benefit both the child and the mother by being as positive as you can, particularly for the mother.” (Grandparent; *Miller et al., 2012*, p. 106)*

### Subtheme 3.3 Family Unit Characteristics

The influence of geographic distances between the nuclear and extended family members was noted by parents and grandparents. Parents reported that extended family members who lived close to them or with them provided more instrumental support than those who lived far away (Casillas et al., 2017; Lee & Gardner, 2015; Mbamba et al., 2023; Sanderson & Aquino, 2023; Shanmugarajah et al., 2022). Some parents described living in a multigenerational household and the grandparents frequently helped with childcare (Lilley et al., 2020). Grandparents sometimes moved to be closer to the nuclear families to provide more support (Miller et al., 2012).

*“We [live] too far away to [help] with the everyday minutia that comes with…the demands and challenges that autism brings.” (Grandparent; *Hillman et al., 2017*, p. 2962)*

Some grandparents reported that family structure influenced their level of involvement. In families with multiple adult children and grandchildren, grandparents were expected to distribute their care and attention equally among their adult children and grandchildren, regardless of the specific needs of each family (Hillman et al., 2017; Miller et al., 2012). This dynamic may result in only grandchildren and their parents receiving more focussed care from the grandparents. One grandmother expressed difficulty in balancing her time: “*well, it is very hard to spread my time equally. They’re almost adding up the hours, my two daughters*” (Grandparent; Miller et al., 2012, p. 107). Concerns about siblings of autistic grandchild were also presented, *“[We make] sure we do not give our other [non-ASD] grandchild more attention because we can interact with him more*” (Grandparent; Hillman et al., 2017).

### Subtheme 3.4 Family Interaction Characteristics

Features of the family relationship and interaction between family members can affect the role extended relatives play in the care for their autistic relatives. This subtheme is further decomposed into two subcategories comprising the family relationship quality and role boundaries.

#### Family Relationship Quality

The quality of the family relationship and communication between family members can impact the contact and closeness of the relationship between autistic individuals and their extended family members (D’Astous et al., 2013; Lee & Gardner, 2015; Mirfin-Veitch et al., 1997). Parents in families lacking warmth and trust before the diagnosis of autism rarely sought help from extended family members (LaRoche & Rivières-Pigeon, 2022; Mbamba et al., 2023). Conversely, parents in close-knit families would actively permit interactions between extended family members and the autistic children. A grandmother commented that, “*[grandson’s parents] encourage their relationship with him [grandson] by calling and asking for our help, and we never say no*” (D’Astous et al., 2013, p. 140).

For involved extended family members, there generally was a climate of open communication and appreciation, facilitating their support (D’Astous et al., 2013; Lu et al., 2022). A grandparent noted she felt her adult child appreciated the “Grandma letters” she wrote to her grandchild, “*I have a great relationship with my daughter, and she appreciates what I do for her*” (D’Astous et al., 2013, p. 140).

Poor communication was frequently cited in the form of argument (Huang et al., 2023; Miller et al., 2012), neglect (Mbamba et al., 2023), and a lack of emotional expression (Lee & Gardner, 2015), which increased tensions in families and impacted the involvement of extended family members negatively.

*“The atmospheres were intense and not good for the child and me...Our family atmosphere had been in a low state for almost two years. The whole family blamed each other, criticized each other, grumbled at each other, and complained about each other. We had remained in this state for two years.” (Parent; *Huang et al., 2023*, p. 11)*

It was common for parents and grandparents to have different opinions on standards of discipline and care when grandparents helped with childcare (Hillman & Anderson, 2019; Woodbridge et al., 2011; Yang et al., 2018). Grandparents often reported having to navigate the tensions arising from differences in opinion by asking parents to clarify their rationale, as one grandparent demonstrated saying, “*I think I’ll understand better why you’ve made the decisions you’ve made for him, if you’ll explain … educate me*” (Grandparent; Yang et al., 2018, p. 368).

#### Family Role Boundaries

Some studies suggested that clear family role boundaries enabled parents to feel supported rather than intruded upon (D’Astous et al., 2013). Some grandparents clearly defined boundaries between the role of the parent and grandparent (Miller et al., 2012; Prendeville & Kinsella, 2019; Yang et al., 2018). Parents also recognised extended family members may have their priorities and avoided relying too heavily on them (Lilley et al., 2020; Mirfin-Veitch et al., 1997).

*“I really value my relationship with my daughter…even though I may not agree with the choices she’s made for her child, I don’t want to become an adversary…I want to be part of the team…she’s the parent; I’m the grandparent. I’m not trying to be the parent.” (Grandparent; *Yang et al., 2018*, p. 367).*

However, many grandparents found themselves struggling to find a balance of support in their adult children and grandchildren’s lives while maintaining independence (Margetts et al., 2006; Miller et al., 2012; Yang et al., 2018). Grandparents described how they put retirement plans on hold, moved house to be closer, and reduced their work hours to better support their grandchildren and adult children (Miller et al., 2012).

*“My husband wants to retire....he wants to go away from the coast, I just said to him [daughter’s] situation is so difficult, she needs the back-up and I’m not prepared to go just yet. I do see myself as a hands-on grandparent to help her wherever I can.” (Grandparent; *Miller et al., 2012*, p. 106)*

In four studies, parents expressed stress related to too much involvement from grandparents in decision-making about their children’s lives (Bai et al., 2024; Lee & Gardner, 2015; Mirfin-Veitch et al., 1997; Wang et al., 2023).

*“My father-in-law tended to decide by himself about important decisions related to my child. He wants us to follow his decisions and educate my child with the methods he supports. We used to go with his decisions at first since we had not raised a child, especially a child with a disability. His behaviours became stressful rather than being helpful as time went on because it is I, the mother of my child, who know the most about my kid and am able to decide. I now wish he could observe by my side and let me decide important issues related to my kid.” (Parent; *Lee & Gardner, 2015*, p.216)*

### Subtheme 3.5 Cultural Influences

Cultural contexts shape the patterns of support provided by extended family members. This subtheme contains two categories: the positive and negative influences of interdependence in family-oriented culture, and the influential role of the elderly.

#### The Positive and Negative Influence of Interdependence in Family-Oriented Culture

Papers reporting on studies conducted in Asian, African, Latino, Spanish, and Aboriginal and Torres Strait Islander families emphasised the influence of a culture of high family interdependence on family relationships. The study authors highlighted that the Latino cultural concept of *Familism* (Casillas et al., 2017; DuBay et al., 2018), Spanish concept of *Familism* (Baena et al., 2024), Ethiopian concept of *Yilunta* (Tekola et al., 2020), the concept of *Kinship support* present in Ghana (Mbamba et al., 2023), Chinese and Korean concept of *Confucian* (Huang et al., 2020; Lee & Gardner, 2015), and the concept of *kinship obligations* present in Aboriginal Australia (Lilley et al., 2020) were associated with family-oriented culture that highlight the importance of interdependence within families.

The strong emphasis on interdependence among family members means that they were expected to support each other, sharing responsibilities such as childcare. For instance, a Latina mother noted the close relationship with extended family members and how family members depend on each other for support:

*“The Latino family is very united. I’m from a Latino family, we’re very close. We communicate. We always tell each other everything. For example, if a sibling has a problem, everyone helps. In my case, my, all my siblings, on my side, everyone knows my daughter’s diagnosis. And everyone understands me, and everyone has supported me, and everyone has helped me. I’m happy with the support I’ve had from my family.” (Parent; *Dubay et al., 2018*, p. 1630)*

Nevertheless, not all aspects of the family-oriented culture inspired support from extended family members. Family-oriented culture was often associated with shame because people tended to be sensitive about other’s evaluations and care about their personal and family social identity. Having an autistic child was considered shameful and damaging to the family reputation among East Asian, African, Aboriginal, and Torres Strait Islander families (Huang et al., 2011, 2020; Lee & Gardner, 2015; Lilley et al., 2020; Tekola et al., 2020). The concerns for the family’s reputation led to a lack of support and stigmatisation from extended family members (Huang et al., 2011, 2020; Lee & Gardner, 2015; Tekola et al., 2020).

*“My mother, I have told you, she used to say take him [my child] to the back of the house when someone comes to the house. And my biggest fight with my mother was: ‘why do you say that; it is not good’. They only think about the family [name] ….” (Parent; *Tekola et al., 2020*, p. 4)*

#### The Influential Role of the Elderly

The influential position of elderly people within the family was also emphasised in studies conducted in China and Korea, where Confucian traditions prevail (Huang et al., 2011; Lee & Gardner, 2015). In Confucian culture, the grandparents were considered the head of the family, and people were expected to respect their parents (Lee & Gardner, 2015). Regardless of whether grandparents accepted or did not accept their grandchild’s disability, they often felt entitled to participate in decision-making related to their adult child and grandchild. Challenging grandparents’ authority in the family could lead to a loss of family support (Huang et al., 2011).

*“I wanted to bring him (child) to the hospital for an examination, but my mother thought that my son’s development was just a little bit late for his age and he would be fine when he grew up. She couldn’t accept that my son has a deficit. I just kept waiting to avoid arguing with my mother, so we didn’t go to hospital for an examination.” (Parent; *Huang et al., 2011*, p.244)*

Although Western research does not directly explain the position of grandparents within the family, several grandparents in western contexts considered themselves facilitators of family communication, informing other relatives about their grandchildren’s autism diagnosis (Prendeville & Kinsella, 2019; Woodbridge et al., 2011). They also acted as disseminators to address the misunderstandings within families (Miller et al., 2012). This fostered a feeling of ease for both autistic individuals and their parents when interacting with other relatives (Woodbridge et al., 2011).

*“I suppose like a typical Irish family you tell your parents and they become the messengers then for everybody else and that’s the way it would have been I suppose.” (Parent; *Prendeville & Kinsella, 2019*, p. 743)*

## Discussion

This systematic review of 42 studies is the first comprehensive synthesis of the role of extended family members, including grandparents, aunts, uncles, and cousins, in the lives of autistic individuals and their parents. Our qualitative synthesis resulted in three themes, describing the types of support provided by extended family members; concerns relating to unhelpful or lack of support offered by extended relatives, and factors associated with the role played by extended relatives. This meta-synthesis has enriched and updated the rapid review by Hillman (2007) on the role of grandparents of autistic children by providing novel information reflecting perspectives of parents and grandparents on the role played by extended family members across cultures and identifying the factors influencing this role. Incorporating views and experiences of parents and grandparents allows a more comprehensive understanding of the role of extended family members to be considered in supporting families with autistic individuals.

Identified types of support in this review includes emotional, instrumental, financial, and informational support. These findings are in line with previous reviews on the role of grandparents in caring for children with broad categories of disabilities (Lee & Gardner, 2010; Novak-Pavlic et al., 2022). Similarly, grandparents made personal financial sacrifices to help with costs related to disabilities and childcare, assisted in diagnosis of disabilities and further help-seeking, and provided emotional support. This review further suggests that other extended family members, such as autistic children’s aunts and uncles, also contribute to providing these types of support. In contrast to supporting neurotypical children, providing childcare to autistic individuals requires additional instrumental, emotional, and financial costs (Mitchell, 2007). For instance, some grandparents of autistic grandchildren acted as primary caregivers due to the substantial parenting demands placed on nuclear families (Huang et al., 2020). Additionally, they provided support in managing the additional challenges associated with the behaviours and social communication of their autistic grandchildren (Woodbridge et al., 2011; Yang et al., 2018). As neurotypical children become more independent with age, the care role of grandparents may decrease over time (Duflos & Giraudeau, 2022). This contrasts with the caregiving role for grandparents of autistic children, where care responsibilities are more likely to persist and may even intensify.

Not all extended family members have the capacity to provide support. Our review described both parents’ neutral experience associated with lack of support as well as the negative experiences from extended family members. This review consistently identified extended family members’ misunderstanding of autism, including the cause of autism, medical and behavioural management, and the current condition of autistic individuals. As a previous review suggested (Novak-Pavlic et al., 2022), extended relatives may have more difficulty in accepting autism than physical disabilities, due to the absence of physical impairments in affected children. Our review advances the previous review by providing novel insights into parental experience associated with negative attitudes and discriminatory behaviours, including blame, judgement, and rejection from extended family members. Consequently, some parents choose not to disclose the diagnosis to their relatives or to self-isolate to avoid such discrimination.

Previous research on grandparents caring for neurotypical children indicates that parents can experience stress arising from excessive control from grandparents, differing child-rearing attitudes, and communication barriers (Hoang & Kirby, 2020). Aligning with previous reviews on disabled individuals (Hillman, 2007; Lee & Gardner, 2010; Mitchell, 2007), our review indicated that parents of autistic individuals faced additional challenges if extended relatives were unable to understand their child’s disabilities.

The findings of this review identified multiple characteristics of autistic individuals, parents, extended family members, and family interaction that might influence the role of extended family members. Factors, such as understanding of autism, positive attitudes, high family relationships quality, and a clear mutual understanding of the boundaries of the role of extended relatives, all contribute to positively perceived support from extended relatives. These findings also suggest positive mechanisms for interventions to improve the involvement and support provided by extended relatives. Interventions targeting extended family members’ understanding of autism and supporting positive interactions between autistic individuals and extended relatives, may increase relative’s knowledge and attitudes towards autism and subsequently the support they are able to offer to autistic children and their parents. Thornicroft et al. (2022) emphasised the importance of psychoeducation and social contact in reducing interpersonal stigma. The evidence of this review supports future initiatives aimed at fostering understanding and acceptance of autism among extended family members.

This review provided insights into the influence of culture on the role played by extended family members. Papers from Asia, Africa, Latin America, and Spain highlighted the importance of family, and in these family-oriented cultural contexts, extended family involvement appeared more evident. Similarly, quantitative studies in America revealed that African Americans and Black Caribbeans received instrumental support from extended relatives more frequently than non-Latinx Whites, regardless of income level (Taylor et al., 2022). This emerging body of research aligns well with a wider body of research on the cultural influence on grandparental childrearing of neurotypical children (Chan et al., 2022; Duflos & Giraudeau, 2022; Sadruddin et al., 2019), indicating variations in the cultural expectations of grandparental involvement in the childcare of autistic children. Conversely, our review also suggests the emphasis on family reputation within family-oriented cultures may hinder acceptance of autism by extended family members. The influential role of grandparents, particularly evident in certain cultures like East Asian (Lee & Gardner, 2015), extends their role beyond conventional caregiving. As key influencers, grandparents may have ability to shape attitudes and foster a more inclusive family environment, thereby influencing other family members’ acceptance of autism. While these studies were important in highlighting broad cultural differences in the role of extended relatives, research to date has not yet fully explored more nuanced dynamics, for example regional or intergenerational cultural differences within a country context (de Leeuw et al., 2020). Therefore, care should be taken against simplistic cultural generalisations and to also consider individual and family characteristics.

## Future Research and Clinical Implications

This review highlighted key aspects related to facilitating the adaptation, support, involvement of extended family members. These findings can inform practitioners in how they support families foster active engagement of extended family members in the care of autistic individuals.

A clear gap identified in this review is the lack of research on the perspectives of autistic individuals themselves in support provided by extended relatives. Moreover, the majority of papers focus on the role of grandparents, with paucity in research focussing on other extended relatives such as aunts, uncles or cousins; and of extended family members who are serving as custodial caregivers, for instance, relatives caring for autistic children left behind when their parents work away from home (Zhu et al., 2023). Recommendations highlighting future research directions (R), priorities for intervention development (I), and clinical practices (P) are provided in Table 3. These are based on key components of family system theory (Turnbull et al., 2010) and align with influencing factors identified in theme 3.

**Table 3:** Recommendations for future research and clinical directions

Levels	Recommendations
Level 1: Family transition and journey towards acceptance	R. Explore how the support needs of families with autistic children change over time and the adjustments extended relatives may need to make to support these evolving needs
	I. Develop the longitudinal interventions assisting extended relatives in adapting to the evolving needs of families with autistic children
	P. Involve extended relatives when supporting families in the journey towards acceptance and changes in school, therapy, or routine, if appropriate
Level 2: Individual	R. Explore views and experiences of autistic individuals, custodial relatives, and extended relatives other than grandparents
	I. Develop and evaluate interventions to address stigma and unhelpful beliefs among extended family members, and establish peer support groups for extended relatives
	P. Provide psychoeducation to support extended relatives’ own needs and concerns. Existing examples, such as, Zakirova-Engstrand et al. (2023)
Level 3: Family unit and cultural influences	R. Explore within-culture strategies to mitigate the effects of unhelpful cultural values or norms. E.g. strategies that might promote support over saving face
	I. Develop and evaluate interventions to tap into positive cultural factors and mitigate negative cultural dynamics. For example, involve extended relatives in intervention delivery when they serve as co-caregivers or families expect their participation (Chlebowski et al., 2020; Lee et al., 2025); empower parents to negotiate co-parenting experiences without violating filial duties or dismissing grandparents’ roles, by using nonviolent communication skills (Hoang et al., 2022)
	P. Recognise the importance of family dynamics and decision-making processes within different cultural contexts. Involve the family elders in the discussion and planning, if appropriate
Level 4: Family interaction	R. Explore how family relationships, communication and role flexibility influence the role of extended family members
	I. Develop and evaluate interventions to improve the quality of family interaction between parents and extended family members
	P. Train families in strategies to improve interactions, and reduce and resolve conflicts among extended relatives and parents

*R* indicates future research topics;* I* indicates opportunities for future intervention development;* P* indicates recommendations for clinical practices

## Limitations

The research presented here should be interpreted in light of some limitations. We excluded articles that did not explicitly include the keywords associated with “extended family” in their research aims, objectives, or as a theme or subtheme in the results or findings. This decision was made because some studies used the term “families” to refer to extended relatives, making it difficult to distinguish whether the views were on extended family members, or on those of nuclear family members. This approach may have resulted in the exclusion of literature about extended family members.

While we did not restrict the language of publication, our search terms were in English, possibly limiting our retrieval of relevant literature without English abstracts and keywords. However, we were able to identify a study in French and one in Chinese through database search. We also used subject headings and conducted forward and backward citation checks to mitigate this limitation. To ensure that relevant Chinese literature was not overlooked, the first author conducted searches using Chinese keywords in Chinese databases, such as CNKI and WanFang, during the preliminary searches stage.

## Conclusion

This study provides a comprehensive and up to date review of the qualitative literature on the role of extended family members in the care of autistic individuals. It identifies factors influencing the role of extended family members at family individual, unit, interaction, and transition levels. To effectively facilitate the active involvement of extended family members, this review recommends evaluating extended family members’ availabilities and abilities to provide support, enhancing the understanding of autism among extended family members, improving the quality of family interactions, and increasing awareness among professionals of the cultural background of families with autistic individuals. In conclusion, a family system approach to support the involvement of extended family members is advocated by this review to lead to a unified family capable of navigating the unique challenges associated with autism.

## Supplementary Information

Below is the link to the electronic supplementary material.

Supplementary file1 (DOCX 20 KB)
