# Supplementary material for: The Role of Extended Family Members in the Lives of Autistic Individuals and Their Parents: A Systematic Review and Meta-Synthesis
Source: Clin Child Fam Psychol Rev. 2025 May 20;28(2):507–39. doi: 10.1007/s10567-025-00525-7 (PMC12162707; doi:10.1007/s10567-025-00525-7)
Supplement: Supplementary file 1 — Supplementary file1 (DOCX 20 KB) [file 10567_2025_525_MOESM1_ESM.docx]

Supplementary Material A: Search terms

This review uses the Sample, Phenomenon of Interest, Design, Evaluation, Research type (SPIDER) tool to guide the search strategy (Cooke et al., 2012).

| 1 | S-Sample- individuals and primary caregivers affected by autism | "development* disorder*" or "development* disabilit*" or "neurodevelopment* disorder*" or "neurodevelopment* disabilit*" or autis* or Asperger or "pervasive develop*" or PDD or ASD |
| --- | --- | --- |
| 2 | PI-Phenomenon of Interest- the role of extended family members | famil* or "extended famil*" or "extended relative*" or household* or "blended famil*" or kinship or "relative care*" or "secondary caregiver*" or grandparent* or grandmother* or grandfather* or "grand parent*" or "grand mother*" or "grand father*" or grandpa or grandma or grandmaternal or grandpaternal or "grand maternal" or "grand paternal" or "mother in-law" or "father in-law" or "daughter in-law" or "son in-law" or grandchild* or granddaughter* or grandson* or "grand child*" or "grand daughter*" or "grand son*" or aunt* or uncle or niece or nephew or cousin* or "family structure" or "co residence*" or "co habit*" |
| 3 | D-Design- Methodologies | interview* or focus group* or survey* or narrative* or "open question*" or "grounded theory" or phenomenological or phenomenal or "thematic analysis" or observ* or ethnograph* |
| 4 | E-Evaluation- lives of individuals and their primary caregivers | experience* or perception* or perspective* or attitude* or believe* or belief* or insight* or characteristic* or opinion* or understand* or feeling* or issue* or knowledge or view* or react* or role* or benefit* or difficult* or contribut* or support* or involve* or intervention or care* or caring or daycare or raising or adapt* or reject* or stigma or shame or discriminat* or isolat* or coping or burden or stress or detect* or diagnosis or recognition or assessment or treatment or evaluat* |
| 5 | R-Research type | qualitative or "mixed method*" |
| 6 | 1 AND 2 AND 4 |  |
| 7 | 3 OR 5 |  |
| 8 | 6 AND 7 |  |

Supplementary Material B: Inclusion and exclusion criteria

| Included | Excluded |
| --- | --- |
| Primary research studies | Literature reviews, conference presentation, protocols, editorials, book chapters, or documents not directly reporting primary research studies |
| Qualitative studies or mix-method studies with a discernible qualitative component | Mixed-method studies where the qualitative part lacked enough details to assess its eligibility. |
| Studies focusing on autism, or a wider range of developmental disabilities as long as at least some of the individuals included in the studies have a diagnosis of autism. For studies reporting diagnosis that include physical conditions rather than autism, only extract specific aspects relevant to the role of extended family members. | Studies primarily focusing on individuals with typical development and the role of extended family members in childcare more generally. |
| Studies reporting on the role of extended family members like grandmothers, grandfathers, uncles, aunts, and cousins, irrespective of the informants (i.e. autistic individuals of all ages, parents, extended family members themselves, etc) | Studies focusing on the role of siblings in caring for individuals with autism, as siblings belong to the nuclear family. |
| Studies about the role of extended family members in the lives of autistic individuals and their primary caregivers, both within the context of general involvement in caregiving and specific involvement in early detection, diagnosis and intervention for autistic individuals |  |
| The role of extended family members must be identified as an aim, research question, or key theme in the results | Studies centred around general parenting experience in raising autistic individuals without a distinct finding related to extended family members. |

Supplementary Material C: Existing main theories and frameworks that informed the theme development in our synthesis

| Extending Theories & Frameworks | Core Elements in Existing Theories and Frameworks that informed the thematic synthesis presented in this review | Themes and Subthemes developed in this review |
| --- | --- | --- |
| Family Support Model  (Kyzar et al., 2012) | First, emotional support, refers to support aimed at enhancing psychosocial well-being by reducing stress and fostering positive emotional feelings. Second, physical support focuses on enhancing the physical health and assisting family members with a disability in acquiring daily living skills. Third, instrumental support indicates assistance aimed at enhancing access to sufficient financial resources and facilitating the completion of essential tasks. Fourth, informational support involves enhancing knowledge and improving decision-making. | Theme1: Types of support  1.1 Emotional support  1.2 Instrumental support *^a^*  1.3 Financial support *^b^*  1.4 Informational support |
| Interpersonal Stigma framework (Thornicroft et al., 2020) | Interpersonal stigma includes three separate but related components: a) knowledge, refers to a lack of knowledge about conditions, or ignorance arise due to misinformation; b) attitudes, refers to negative emotional reactions towards people with conditions; c) behaviours, refers to discrimination and exclusion against towards people with conditions. | Theme 2: Unhelpful or lack of support  2.1 Misunderstanding about autism  2.2 Absence of support with caregiving *^c^*  2.3 Negative attitudes and discriminatory behaviours |
| Family System Theory  (Turnbull & Turnbull, 2010) | The family unit is a dynamic system, and family members have reciprocal effects on one another. Family System Theory includes four dimensions: a) Family characteristics include the Characteristics of family unit (e.g. Family size and form, Cultural background, Socioeconomic status, Geographic location); Individual characteristics (e.g. Severity of disabilities; Health status); and Unique circumstances. b) Family interactions refer to the interactions amongst the marital, parental, sibling and extended family subsystems. c) Family functions are the outcomes of family characteristics and interactions. d) Family life cycle represents the changes of the family experience over time. | Theme 3 Factors influencing the role of extended family members  3.1 A journey towards acceptance of autism  3.2 Characteristics of individual family member  3.3 Family unit characteristics  3.5 Cultural influences |
| The Circumplex Model  (Olson et al., 2019) | The Circumplex Model organises family dynamics into three dimensions: Cohesion (the emotional bonding between family members); Flexibility (the amount of change in family leadership, role relationships and boundaries); Communication (the listening skills, speaking skills, self-disclosure, clarity, continuity tracking, respect and regard of the family as a group) | Theme 3 Factors influencing the role of extended family members  3.4 Family interaction characteristics |

*a.* Kyzar et al.’s (2012) physical support was included in subtheme “instrumental support” due to its practical nature, as revealed through data analysis.

*b.* We differentiated “financial support” from Kyzar et al.’s (2012) instrumental support, based on the data indicating that financial support is material-focused rather than practical in nature.

*c.* Given parents’ neutral experience associated with lacking support identified through data analysis, a further layer “Absence of support with caregiving” was added here.
